# Use of diverging apertures to minimize the edge scatter in passive scattering proton therapy

**DOI:** 10.1120/jacmp.v16i5.5675

**Published:** 2015-09-08

**Authors:** Tianyu Zhao, Bin Cai, Baozhou Sun, Kevin Grantham, Sasa Mutic, Eric Klein

**Affiliations:** ^1^ Department of Radiation Oncology Washington University School of Medicine St. Louis MO USA

**Keywords:** proton, passive scattering, aperture, edge scatter

## Abstract

The purpose of this study was to evaluate the use of diverging‐cut aperture to minimize collimator contamination in proton therapy. Two sets of apertures with nondivergent and divergent edge were fabricated to produce a 10 cm×10 cm field at the radiation isocenter of a single‐room proton therapy unit. Transverse profiles were acquired in a scanning water tank with both aperture sets. Up to 9.5% extra dose was observed from aperture scattering near the field edges with the nondivergent aperture set at 2 cm above the water surface and remained 3.0% at depth of 10 cm. For the divergent set, the contamination was reduced to less than 3.5% and 1.3%, respectively. Our study demonstrated that scattering from apertures contaminated the dose distribution near the field edge at shallow depth. A diverging‐cut aperture was capable of reducing the contamination and is recommended for use in passive scattering proton therapy, especially when critical organs are lateral and proximal to the target at shallow depth.

PACS numbers: 87.55.ne, 87.56.nk

## I. INTRODUCTION

Aperture in proton therapy is modeled as an infinitesimally thin layer of beam stopper, although the core algorithms of all commercially available treatment planning systems (TPS) has evolved from ray‐tracing to pencil beam, which are more physically meaningful.^(1)^ It remains a convention to mill an aperture with nondiverging cut on the inner surface that encompasses the open field. It is straightforward for a machine shop to create a 2D machine file directly on the output file from TPS. One of the consequences from such a simplification is that it introduces regions where protons interacting with the aperture are not included in the TPS does calculation at all,^2^, ^3^ even though there is a nontrivial probability of these protons reentering patients. These edge‐scattered protons from aperture add unexpected horns on beam profiles, perturbing dose distribution, increasing skin dose, and compromising the plan conformality. This perturbation in dose distribution is regarded as a contamination because of the deviation from the primary protons in terms of energy and direction of motion. The magnitude of this contamination has been underestimated or ignored clinically, as no commercial TPS takes it into consideration.

Aperture scattering has been investigated by several groups theoretically.^4^, ^5^, ^6^ Although those studies aid the understanding of aperture scattering and could be adopted to predict or correct the dose calculations, they have not been implemented in current TPS. In this study, we hypothesized that the aperture scattering would be minimized if the divergence of the inner surface of aperture is in accordance with beam divergence. The beam profiles acquired with divergent and nondivergent aperture were compared to demonstrate the improvement with such a simple solution that no modification of TPS is needed. The guideline on fabricating such a divergent aperture and quality assurance are also discussed.

## II. MATERIALS AND METHODS

**Figure 1:**
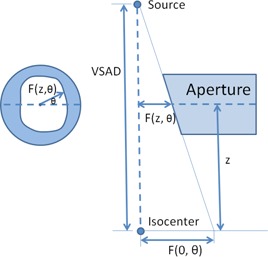
Illustration for Eq. (1) that calculated the radial distance to the central axis from a point on the inner surface.

The milling instruction of divergent aperture is illustrated in Fig. 1. Although the full thickness of brass was provided by a group of slabs (between two and four usually) to reduce the weight of a single slab of aperture, they were treated as an unintegrated set in this study since all slabs shared the same divergence on the inner surfaces. The 3D model of the aperture could be built based on the following generic equation in a cylindrical coordinate system around the central axis pointing to the source from the treatment isocenter. R(z,θ), which denoted the radial distance to the central axis on a transverse plane at a distance of z from the isocenter, could be written as

(1) $$R(z,\theta )=\frac{VSAD-z}{VSAD}R(0,\theta )$$

where *VSAD* was the virtual source–axis distance, and θ∈[0,2π) was the azimuth angle on the aperture. With knowledge of snout position and VSAD, a 3D model of aperture with divergent inner surface in alignment with beam divergence can be easily reconstructed.

Two sets of apertures, one nondiverging cut and one diverging cut, were fabricated to produce a 10 cm×10 cm field at the isocenter. The snout position, which was defined as the distance from the distal surface of the aperture set to the isocenter, was 23.4 cm for both sets. A field of 15 cm in range (R90) and 10 cm in modulation width (R95–R90) was used. The air gaps in our measurements ranged between 13.4 cm and 25.5 cm, depending on the depth of measurement. The same field was also the standard calibration field for IAEA TRS 398 protocol in our clinic.

A microionization chamber (CC04, IBA dosimetry, Nashville, TN) was used to acquire profiles at four depths: 2 cm above the water surface, 2 cm, 7.5 cm, and 10 cm below the water surface, in a water tank (Blue Phantom, IBA Dosimetry, Nashville, TN). All profiles were acquired on transverse plane going through the radiation isocenter, despite at various depths with respect to the water surface. Profiles acquired were compared to treatment planning and a 1D gamma analysis (3%, 1 mm)^(7)^ was performed. Field flatness, defined on two lateral penumbra widths inside the 50% isodose line laterally, and field heterogeneity, defined as the percentage of maximum dose in a field to the dose on the central axis, were reported.

To test our hypothesis that the contamination from aperture edge scattering was minimized when the divergence of the inner surface of aperture was in alignment with the beam divergence, variations in snout positions were deployed deliberately to introduce mismatching of divergence between the beam and the aperture. In our study, three additional profiles were scanned at 2 cm below the water surface with snout positions set at 15 cm, 20 cm, and 25 cm, in addition to snout position of 23.4 cm on which the divergence of the inner surface was fabricated initially.

## III. RESULTS

**Figure 2:**
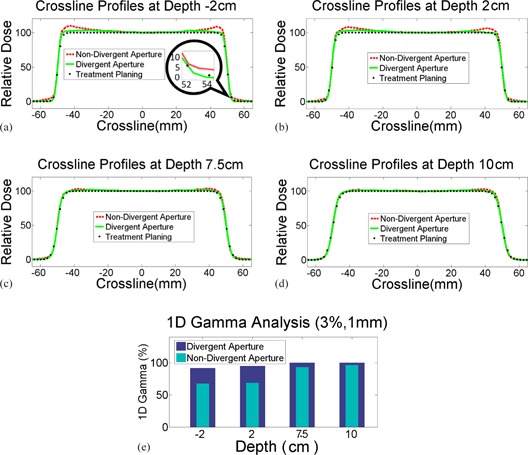
Comparison of crossline profiles of divergent aperture, nondivergent aperture, and treatment planning at various depths. Noticeable differences were observed along the field edges, both inside and outside of the fields. The differences vanished with depth in the water.

Measurements of crossline beam profiles at four depths in the water tank are shown in Fig. 2(a) to (d). Horns were clearly observed near the edges for nondiverging cut aperture sets, as indicated by the red dashed lines. These horns had maximum intensity at shallow depths and gradually faded out with depth. However, the intensity of the horn from the divergent aperture set, even at shallow depth, was much less and closer to the profiles from TPS. The 1D gamma analysis (Fig. 2(e)) showed 91.6% passing rate for the divergent aperture set, versus 67.5% for the nondivergent aperture set, at 2 cm above the water surface. The gamma passing rate increased with depth for both aperture sets, with 95.0% versus 68.5% at 2 cm, 100% versus 93.1% at 7.5 cm, and 100% versus 96.4% at 10 cm below the water surface, respectively. The measurements demonstrated that heterogeneity from aperture scattering was reduced generally with depth, and reduced significantly when switching aperture from nondiverging cut to diverging cut.

**Figure 3:**
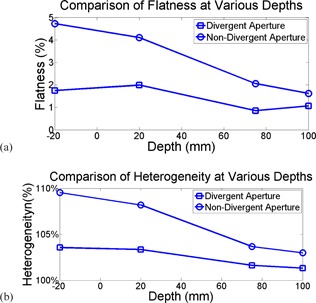
Comparison of crossline flatness (a) at various depths for both divergent and nondivergent apertures. Comparison of maximum heterogeneity (b) in crossline profiles at various depths for both divergent and nondivergent apertures.

A quantitative analysis of flatness of beam profiles is shown in Fig. 3(a). All fields demonstrated a clear trend of flattened profiles with depths. However, for fields under the nondivergent aperture set, the magnitude of the change was dramatic as protons went deeper into water, from 4.7% to 1.6% as the depth changed from 2 cm above the water surface to 10 cm under the water surface. For fields with the divergent aperture set, the flatness dropped from 1.7% to 1.0%, respectively, flatter and more consistent than its nondivergent peer, indicating a significant role of diverging cut in reducing the edge contamination.

Figure 3(b) shows the heterogeneity of the field as a function of depth in water for both aperture sets. The heterogeneity was measured 109.5% at 2 cm above water surface under the nondivergent aperture, and dropped to 103.0% at depth of 10 cm. The use of the divergent aperture set, in comparison, reduced the heterogeneity to 103.5% and 101.3%, respectively.

**Figure 4:**
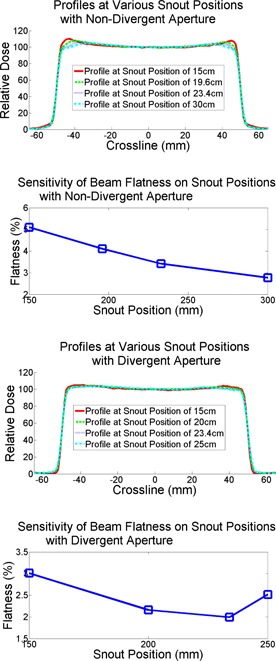
Comparison of sensitivities of profile flatness at 2 cm under the water surface on snout positions for both divergent and nondivergent apertures.

Figure 4 shows the sensitivity of flatness of the divergent aperture set measured at depth of 2 cm for various snout positions. Minimal flatness of 1.8% was found in the scan with the divergent aperture and the original snout position of 23.4 cm, affirming that minimal aperture scattering was achieved only when the beam divergence was in alignment with the inner surface of the aperture.

## IV. DISCUSSION

Our study showed, as reported previously by other groups, that there was a generalized scattering contamination from apertures perturbing dose near the edge of a field. As the contamination faded off with depth, the dosimetric effect was prominent in proximal region, with dosimetric impact mostly severe on skin. In our study, profiles were not acquired on the water surface simply because the rippling of water on the surface would break down the condition of equilibrium for the law of Bragg cavity and cause significant noise in measurements. Instead, we took the measurement at 2 cm above the water surface, which, if ignoring a small correction factor from inverse square law, essentially represented the skin dose. Our measurement showed a local perturbation up to 10% on the dose profiles near the edges. This extra dose was nontrivial when treating without compensator, such as brain fields in craniospinal irradiation, or treating large lesions at shallow depth with full modulation, such as meningioma and chest wall. Most clinical cases come with compensators. The presence of compensator in the bema line was equivalent to adding additional material that pulled back the range of protons in patients up to the maximum water‐equivalent thickness of the compensator. It mitigated the dosimetric effect from aperture scattering. The magnitude of mitigation depended on compensator thickness, target shape, smearing margin, and border smoothing margin — the last two factors of which thinned the compensator below the aperture edge and compromised the mitigating effect.

Milling styles had no impact on field size. However, the radiation fields with the nondivergent aperture set showed a slower lateral gradient at low dose region, as indicated by the zoomed‐in patch in Fig. 2(a). The slow gradient gradually vanished with depth. This observation indicated that some protons, if a nondivergent aperture was used, would penetrate the lower corner of the aperture and add some low‐dose contamination outside of the open field. Use of divergent apertures would eliminate this contamination.

The cost of cutting a divergent aperture is the same as the cost to cut a nondivergent aperture, provided the machine shop has the expertise to program the milling machine to account for the divergence. Quality assurance of a set of divergent apertures might take one extra step to check that the outlines on both surfaces, proximal and distal, agree with the plan sent to the machine shop. For proton machines that do not index the slabs in the snout, the order of aperture slabs might pose an additional safety issue. A simple visual marker could be placed on the outer surface of the aperture set during the QA process and checked by therapists before delivery.

## V. CONCLUSIONS

In this study, we proposed and verified a solution to minimize the dose contamination from aperture scattering on the inner surface by adopting a divergent aperture. This simple solution reduced the heterogeneity in dose distribution and improved the fidelity of dose distribution to treatment plan.

## Supporting information

Supplementary Material

Supplementary Material

Supplementary Material

Supplementary Material

Supplementary Material

Supplementary Material

Supplementary Material
