# Supplementary figures and images for: Use of diverging apertures to minimize the edge scatter in passive scattering proton therapy
Source: J Appl Clin Med Phys. 2015 Sep 8;16(5):367–72. doi: 10.1120/jacmp.v16i5.5675 (PMC5690161; doi:10.1120/jacmp.v16i5.5675)

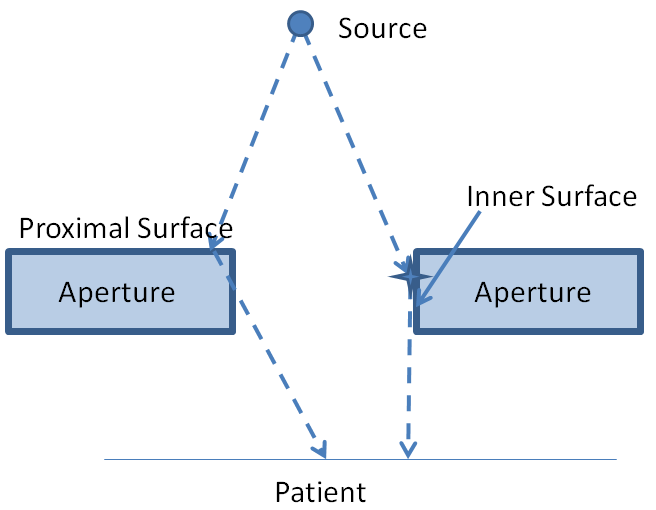

Supplement: Supplementary file 1 — Supplementary Material [file ACM2-16-367-s001.png]

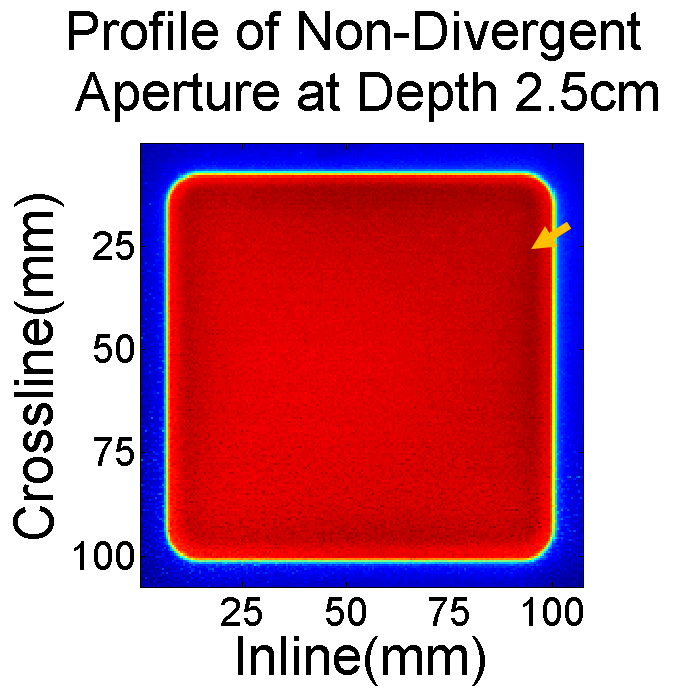

Supplement: Supplementary file 2 — Supplementary Material [file ACM2-16-367-s002.png]

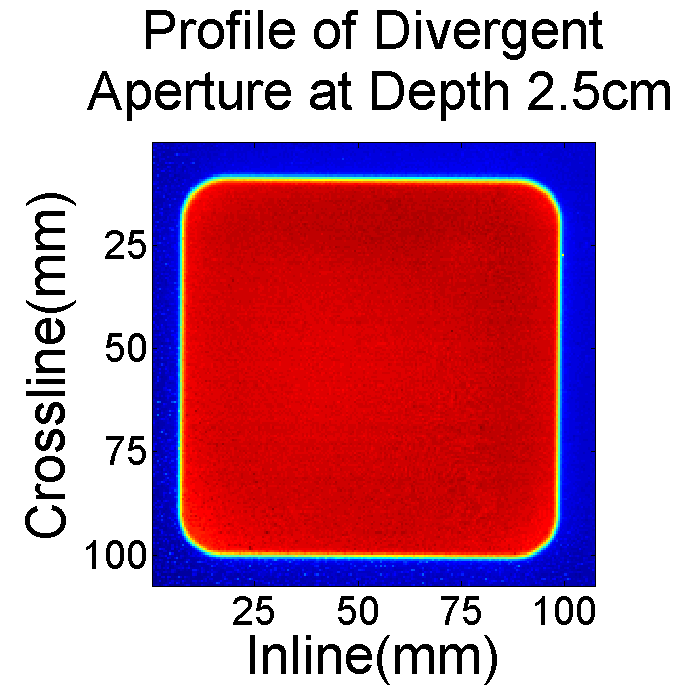

Supplement: Supplementary file 3 — Supplementary Material [file ACM2-16-367-s003.png]

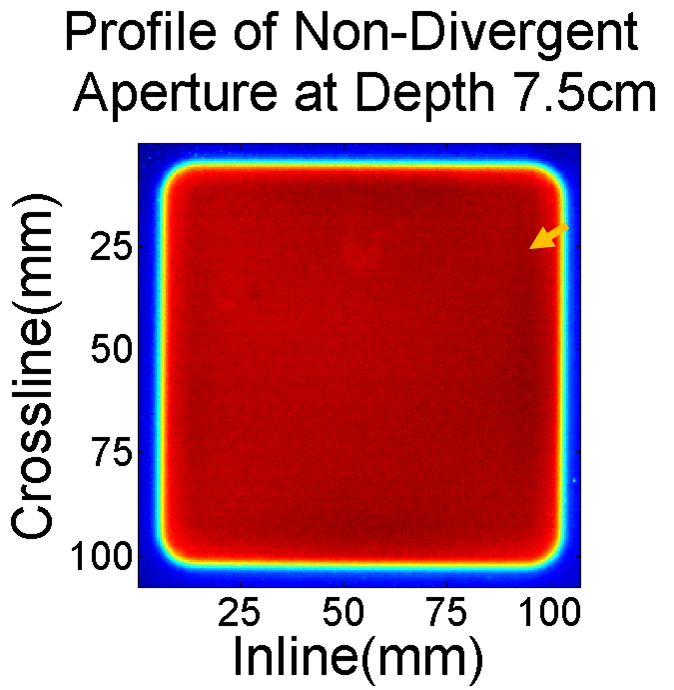

Supplement: Supplementary file 4 — Supplementary Material [file ACM2-16-367-s004.png]

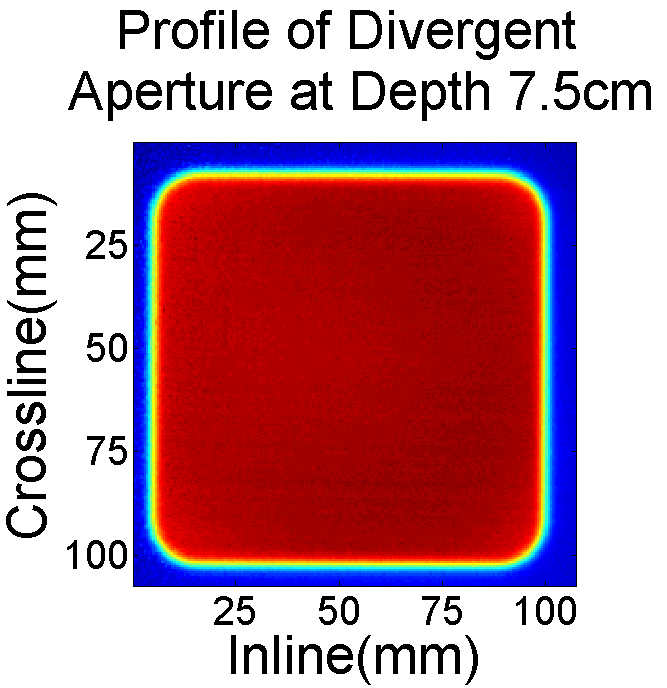

Supplement: Supplementary file 5 — Supplementary Material [file ACM2-16-367-s005.png]

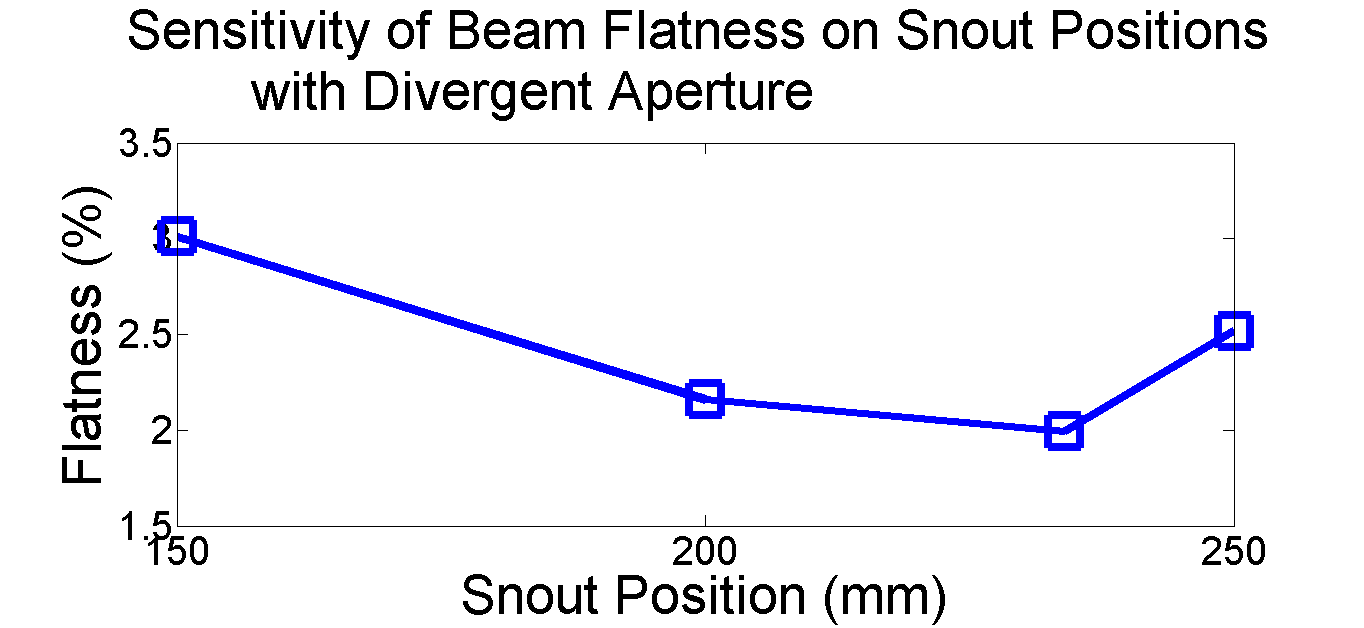

Supplement: Supplementary file 6 — Supplementary Material [file ACM2-16-367-s006.png]

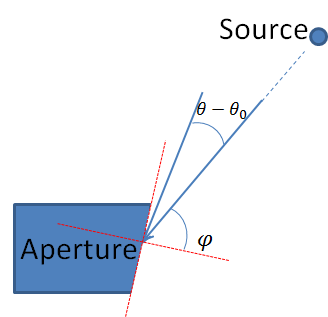

Supplement: Supplementary file 7 — Supplementary Material [file ACM2-16-367-s007.png]
